# Uncertainty analysis of adsorption measurements using commercial gravimetric sorption analyzers with simultaneous density measurement based on a magnetic-suspension balance

**DOI:** 10.1007/s10450-020-00236-1

**Published:** 2020-04-27

**Authors:** Xiaoxian Yang, Reiner Kleinrahm, Mark O. McLinden, Markus Richter

**Affiliations:** 1 Fluid Science & Resources Division, The University of Western Australia, Crawley, WA 6009, Australia; 2 Thermodynamics, Ruhr University Bochum, Bochum 44780, Germany; 3 Applied Chemicals and Materials Division, National Institute of Standards and Technology, Boulder, CO 80305, USA; 4 Applied Thermodynamics, Chemnitz University of Technology, Chemnitz 09107, Germany

**Keywords:** Adsorption isotherm, Gravimetric sorption analyzer, Magnetic-suspension coupling, Tandem-sinker densimeter, Uncertainty analysis

## Abstract

A commercial gravimetric sorption analyzer, which is based on a magnetic-suspension balance, was significantly improved to reduce the uncertainty in adsorption measurements. In a previous paper, we investigated the force-transmission error (FTE) of the instrument’s magnetic-suspension coupling, and we analysed the uncertainty of the density measurement. In the present paper, equations for the determination of the adsorption on porous and quasi non-porous materials are provided, where the FTE is taken into account, and a detailed uncertainty analysis is presented. The uncertainty analysis was applied to both the improved measurement system and a typical commercial gravimetric sorption analyzer. Adsorption test measurements were conducted with carbon dioxide along the *T* = 283 K isotherm at pressures up to the dew-point pressure using both a porous material (zeolite 13X) and a quasi non-porous material (solid metallic sinkers). The major uncertainty contributions for adsorption on the porous material were the mass and volume of the adsorbent sample and the assumption of the density of the adsorbed fluid; for the quasi non-porous material, the main contributions were the weighing values of the balance, the density of the investigated fluid in the gas phase, and the volume of the non-porous material. The influence of the FTE on the adsorption on the porous material was approximately 0.002 mmol·g^−1^, which was negligibly small; but the influence of the FTE was significant in the case of the quasi non-porous material, i.e., approximately 0.7 mmol·m^−2^ or about 22% of the adsorption capacity with the highest adsorption observed in this work (near the dew-point pressure). This indicates that the influence of the FTE increases significantly with decreasing adsorption capacity of the adsorbent sample.

## 1 Introduction

**Fig. 1:**
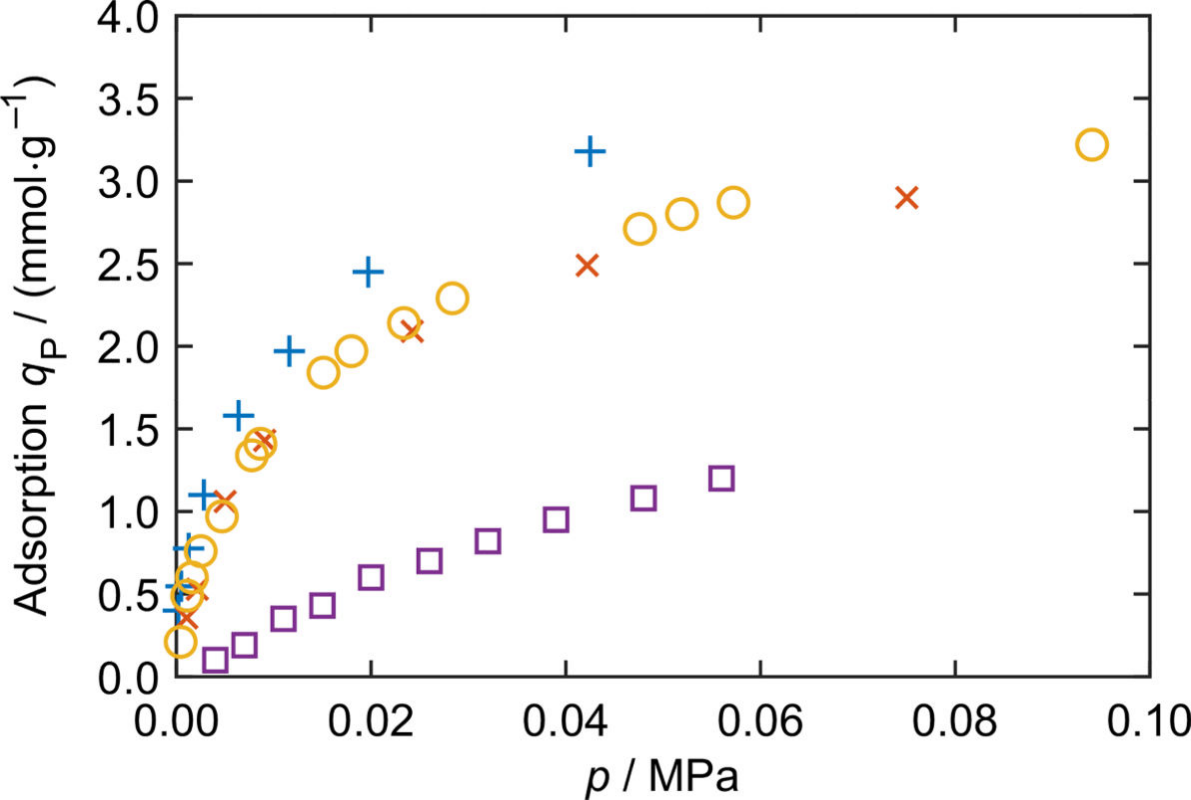
Results of adsorption measurements of carbon dioxide on zeolite 13X at *T* = 323.15 K, conducted by four different research groups: +, Wang and LeVan 2009; ×, Cavenati et al. 2004; ○, Hyun and Danner 1982; □, Deng et al. 2012. The large deviations among the four data sets are briefly discussed in Sect. 1

Gas adsorption and desorption naturally occur in various physical, chemical and biological systems. Technologies based on sorption are widely applied in industries utilizing porous materials like activated carbon, silica gels, metal–organic frameworks, zeolites and many more. Isothermal adsorption measurements of gases on porous materials provide fundamental information for the system design in industrial applications. However, the results of the adsorption measurements of the same gas on the same type of porous material performed by different research groups often show large deviations. For example, Fig. 1 shows the adsorption measurements of carbon dioxide on zeolite 13X along the *T* = 323.15 K isotherm of four different groups (Wang and LeVan 2009; Cavenati et al. 2004; Hyun and Danner 1982; and Deng et al. 2012); their differences are very large. An inter-laboratory study (Zlotea et al. 2009) carried out to evaluate the hydrogen sorption properties of a commercial microporous carbon material showed that the dispersion of isotherms measured by fourteen laboratories increased with pressure with relative deviations up to 36%.

Such large deviations have been commonly realized, and there is increasing research to investigate the reasons and to standardize gas–solid sorption measurements. Kaye et al. (2007) reported that the gas adsorption capacity was significantly affected by the preparation and handling methods of the porous samples, e.g., the differences in the reaction temperature and reaction time (please note: the term “reaction” as stated by Kaye et al. (2007) refers most likely to “regeneration”). Broom and Webb (2017) discussed the potential pitfalls encountered in hydrogen sorption measurement; the pitfalls were grouped into issues associated with instrument design and calibration, sample size, sample and gas purity, isotherm measurement procedure, achievement of equilibrium, and data analysis. Gensterblum et al. (2009) reported the measurement results of an inter-laboratory study among four European research laboratories and showed that when thorough optimization of instrumentation and measurement as well as proper sample preparation procedures were applied, the carbon dioxide sorption on Filtrasorb 400 activated carbon in the supercritical range could be determined accurately with both gravimetric and volumetric instruments. Nguyen et al. (2018) reported the results of an international inter-laboratory study led by the National Institute of Standards and Technology on the measurements of high-pressure excess carbon dioxide sorption isotherms on a reference material (ammonium ZSM-5 zeolite) and provided recommendations for optimising the acquisition of physisorption isotherm data including activation procedures, data processing methods to determine adsorption uptake and the appropriate equation of state (EOS) to be used.

There is no doubt that accurate measurement techniques, strict experimental procedures, deep understanding of the measurement uncertainty, and data analysis methods based on the interdisciplinary knowledge of sorption science and thermophysical properties are of paramout importance in providing reliable adsorption data. The question is how much exactly the large deviation among different researchers results from the measurements and the methods of data analysis? There are various techniques in measuring adsorption isotherms, e.g., by the use of a volumetric adsorption equilibrium apparatus (e.g., Wang and LeVan 2009) or a gravimetric sorption analyzer incorporating a magnetic-suspension balance (e.g., Cavenati et al. 2004). In the present work, we focus on the latter one, and with a detailed uncertainty analysis, we try to answer this question regarding a gravimetric sorption analyzer. Although an uncertainty analysis of a gravimetric sorption analyzer has been presented in the literature (e.g., Ottiger et al. 2008), here, we also include the analysis of the force-transmission error (FTE, see Sect. 3.1) and the analysis for the measurements of nonporous materials in this work.

The usual type of a gravimetric sorption analyzer (see Sect. 2) has been widely used for sorption measurements (e.g., Dreisbach and Lösch 2000; May et al. 2001; Cavenati et al. 2004; and Hefti et al 2015). In our previous work (Kleinrahm et al. 2019), a commercial gravimetric sorption analyzer was significantly improved to reduce the measurement uncertainty. Moreover, the FTE of the magnetic-suspension coupling was systematically investigated, and the uncertainty of the density measurement was analysed in detail. In the present work, equations for the determination of the adsorption on porous and non-porous materials are provided, where the FTE is taken into account. Furthermore, a detailed uncertainty analysis is presented. The improved measurement system was used to conduct adsorption measurements of carbon dioxide on zeolite 13X (porous material) and on solid metallic sinkers (quasi non-porous material) along the isotherm at *T* = 283 K up to the dew-point pressure. The uncertainty analysis was applied to both the improved measurement system now available at Chemnitz University of Technology and to a hypothetical typical commercial gravimetric sorption analyzer.

## 2 Apparatus description

### 2.1 Measurement principle

**Fig. 2:**
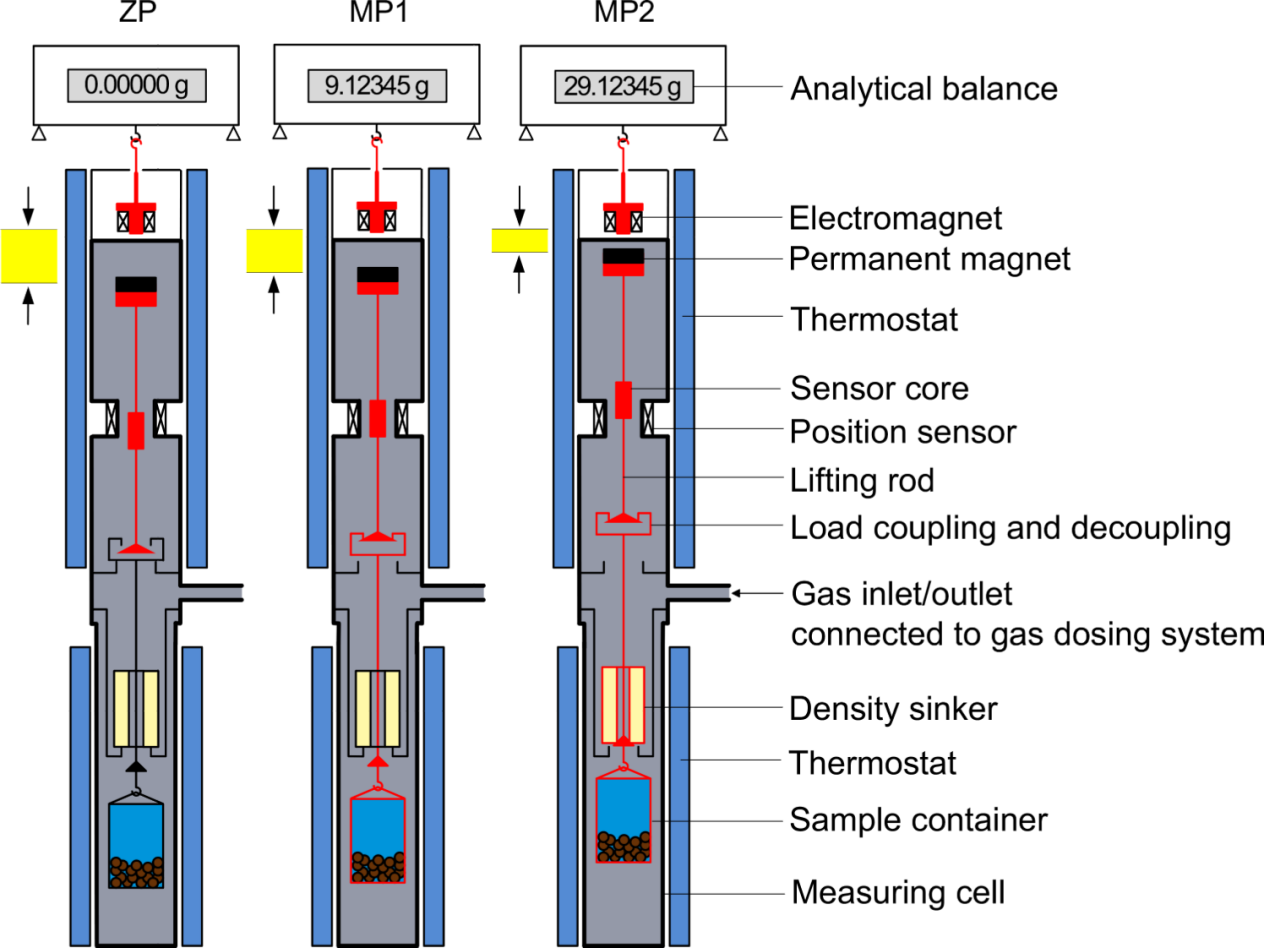
Schematic representation of the three weighing positions of a typical commercial gravimetric sorption analyzer. ZP: zero position or tare position, where only the permanent magnet with the lifting rod assembly is in suspension; MP1: measuring position 1, where the sample container with its lifting rod and the porous sample inside are lifted; MP2: measuring position 2, where the density sinker at the top position is lifted into suspension as well

A gravimetric sorption analyzer incorporating a magnetic-suspension balance was first developed by Dreisbach and Lösch in the late 1990s and published by them in 2000. It was designed to measure the adsorption of a sample gas on a porous material and, by the use of a “density sinker”, to simultaneously measure the density of the sample gas surrounding the porous material. Such an instrument has been commercially available from Rubotherm, Germany, since 1999 (since 2016, the company is a part of TA Instruments, USA).^1^ The principle of a gravimetric sorption analyzer is illustrated in Fig. 2. The core apparatus is normally accommodated in a frame with an analytical balance at the top, a magnetic-suspension coupling underneath and a measuring cell at the bottom. The balance is placed under ambient conditions, while both the magnetic-suspension coupling and the measuring cell are thermostated with an external circulating bath. The measuring cell is connected to a gas-dosing system, which is used to control the pressure inside the measuring cell.

The magnetic-suspension coupling is the key component of a gravimetric sorption analyzer to be used over large temperature and pressure ranges. It comprises (1) an electromagnet that is hung from the weighing hook of the balance, (2) a permanent magnet together with a lifting rod, which are together levitated by the electromagnet, and (3) a position sensor as well as a feedback control circuit that makes fine adjustments in the electromagnet current to maintain the permanent magnet in different stable suspension positions. The change in the height of the permanent magnet yields three stable positions: (1) a tare or zero position (ZP), where only the permanent magnet together with the lifting rod assembly is freely levitated, (2) a lower measuring position 1 (MP1), where the adsorbent (sample container with its lifting rod and the porous material inside) at the bottom position is lifted, and (3) an upper measuring position 2 (MP2), where both the adsorbent at the bottom position and the density sinker at the top position are picked up. The density sinker, as it is named, is used for the determination of the density of the sample gas. By using the magnetic-suspension coupling, the load of the density sinker and the adsorbent in the pressurized measuring cell are transmitted to the balance (through the pressure-proof wall of the measuring cell) without direct contact. Based on the difference in the weighing values in positions MP1 and MP2, the density of the fluid in the measuring cell can be calculated (see Sect. 3.1), and based on the difference in the weighing values in positions ZP and MP1, the adsorption on the adsorbent can be calculated (see Sect. 3.2).

### 2.2 Improved measurement system

**Fig. 3:**
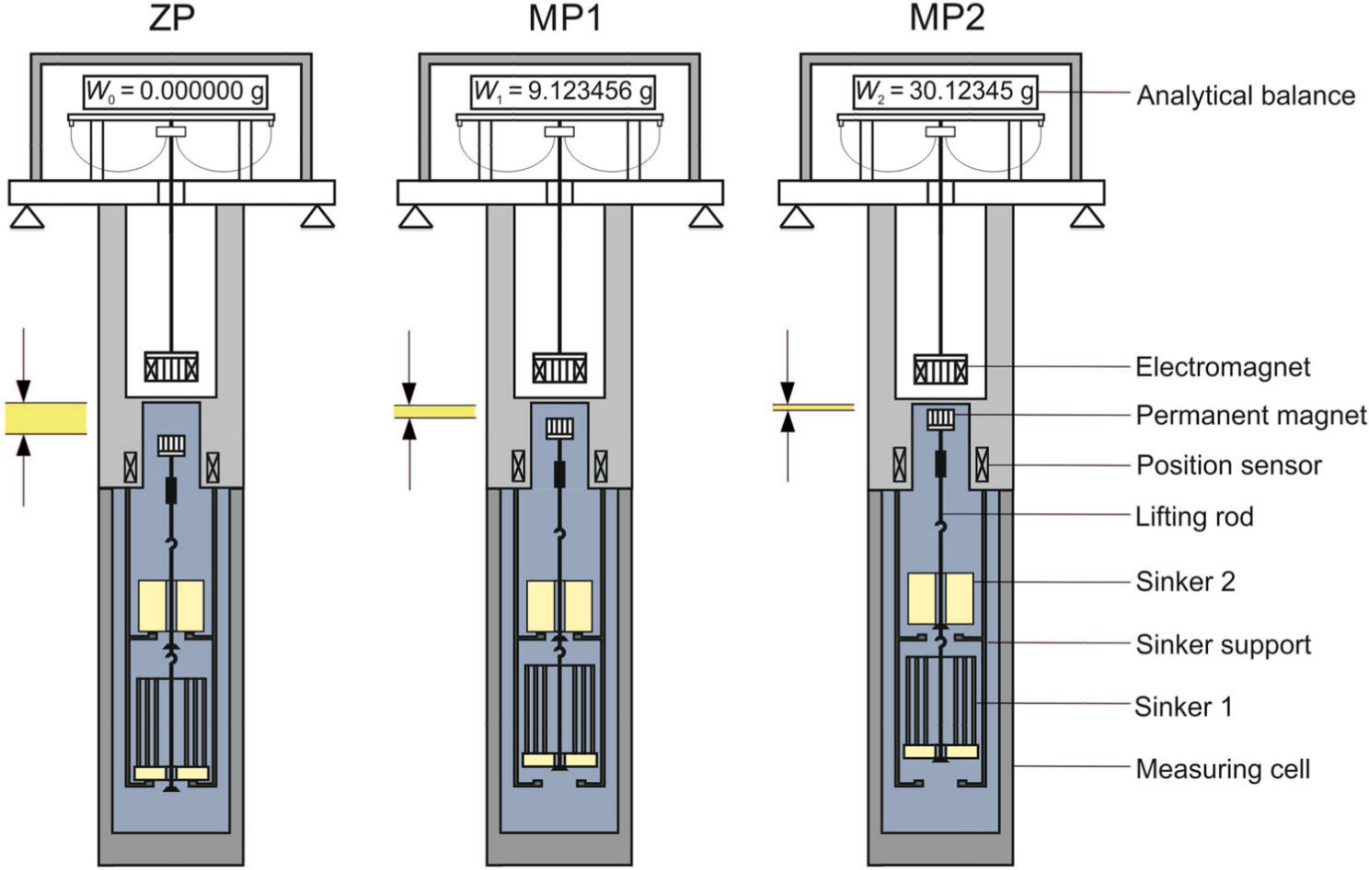
Schematic representation of the three weighing positions of a tandem-sinker densimeter. ZP: zero position or tare position, where only the permanent magnet and the lifting rod assembly is in suspension; MP1: measuring position 1, where sinker 1 is lifted; MP2: measuring position 2, where both sinkers are lifted into suspension

In our previous work (Kleinrahm et al. 2019), we took a gravimetric sorption analyzer and improved it to obtain a significantly lower measurement uncertainty; it was essentially turned into a tandem-sinker densimeter, as we named it in that work. A schematic diagram of the tandem-sinker densimeter is illustrated in Fig. 3. The key modification was the reversible replacement of the sample container at the bottom position to a solid sinker, which has a relatively large surface-to-volume ratio and is named “sorption sinker” here to be distinguished from the density sinker. This improved measurement system is capable of sorption measurements for both porous materials and non-porous materials (e.g., the sorption sinker) with simultaneous density measurement. Detailed information about the improved measurement system and the modifications are described in our previous work (Kleinrahm et al. 2019). Here, we only summarize the key information.

The temperature of the measuring cell was measured with a well-calibrated 100 Ω platinum resistance thermometer (Lake Shore, USA, type: PT-103) in conjunction with a resistance bridge (Anton Paar, Austria, type: MKT50) and the calibrated internal resistor (approximately 400 Ω) of the bridge. The thermometer was calibrated in-house on ITS-90 at the triple point of water (273.160 K), the melting point of gallium (302.9146 K), and the freezing point of indium (429.7485 K). The pressure was measured with a vibrating quartz-crystal-type transmitter (range up to 13.8 MPa, Paroscientific, USA, type: 42 K-101); the transmitter was thermostated at approximately *T* = 333.15 K to avoid condensation of the sample gas. The pressure measurement chain was annually calibrated in-situ with a piston gauge (Fluke Calibration, USA, type: PG-7601). The weighing of the sinker and the adsorbent was conducted with an analytical balance (readability: 1 μg, Mettler-Toledo, Switzerland, type: WXS206SDU) via the magnetic-suspension coupling. The density sinker was a 20 g titanium sinker with a relatively small surface-to-volume ratio and with the surface polished with abrasive. The sorption sinker was made of stainless steel (type 1.4301, according to the European standard) with a mass of about 9.3 g and a relatively large surface-to-volume ratio; the surface of the sorption sinker was sandblasted with 250 μm particles. Detailed information of these two sinkers are summarized in Table 1. The expanded uncertainty (*k* = 2) of the measurement system was estimated to be: 16 mK for temperature, between (0.1 and 0.7) kPa for pressures from vacuum to 8 MPa, and 0.020 kg·m^−3^ in density. The systematic error due to the FTE has been included in the uncertainty of density measurement. The combined expanded uncertainty (*k* = 2) in density measurements of pure fluids, with the exception of measuring points in the vicinity of the dew point and the critical point, is 3.8 × 10^−4^*ρ* or 0.024 kg·m^−3^, whichever is larger.

## 3 Working equations

### 3.1 Determination of the fluid density and explanation of the force-transmission error

The magnetic-suspension coupling transmits the loads of the density sinker and the adsorbent (container with porous material or sorption sinker) in the pressurized measuring cell to the balance, which is placed under ambient conditions. However, since neither the coupling housing nor the sample gas are completely magnetically neutral, a small systematic FTE occurs. To obtain the highest achievable accuracy, the FTE caused by the magnetic-suspension coupling of the improved measurement system was systematically investigated in our previous work (Kleinrahm et al. 2019). Here, we only summarize the key information.

According to the Archimedes (buoyancy) principle, the readings of the analytical balance at the positions MP1 (*W*_1_) and MP2 (*W*_2_) reflect the mass *m*_S_ and volume *V*_S_ of the density sinker and the density of the fluid *ρ*_fluid_ in the measuring cell. The weighing results yield:

(1) $${\left(W_{2}-W_{1}\right)}_{\text{fluid}\;}=\left(m_{\text{S}}-\rho_{\text{fluid}\;}\cdot V_{\text{S}}\right)\cdot \alpha \cdot \phi_{12},$$

where *α* = (1 − *ρ*_air_/*ρ*_calib_)^−1^ is the balance calibration factor with *ρ*_air_ being the air density in the laboratory and *ρ*_calib_ being the density of the calibration mass in the balance. The value *ϕ*_12_ is the coupling factor, which accounts for the FTE (due to the change in height of the permanent magnet) between positions MP1 and MP2. The coupling factor *ϕ*_12_ can be divided into two parts, an apparatus contribution, *ε*_vac,12_, and a fluid contribution, *ε*_fluid,12_; the relation is:

(2) $$\phi_{12}=1+\varepsilon_{\text{vac},12}+\varepsilon_{\text{fluid},12}$$

The value *ε*_vac,12_ should be calculated from the result of a measurement with the measuring cell evacuated, before or after an isothermal measurement of a fluid:

(3) $$\varepsilon_{\text{vac},12}=\frac{m_{\text{S},\text{vac}}^{*}}{m_{\text{S}}}-1,$$

where m^*^_S,vac_ = (*W*_2_ – *W*_1_)_vac_/*α*. The value of *ε*_fluid,12_ is approximately proportional to the specific magnetic susceptibility *χ*_s_ and the density *ρ*_fluid_ of the sample fluid as demonstrated by McLinden et al. (2007):

(4) $$\varepsilon_{\text{fluid},12}=\varepsilon_{\rho ,12}\cdot \frac{\chi_{\text{s}}}{\chi_{\text{s}0}}\cdot \frac{\rho_{\text{fluid}\;}}{\rho_{0}},$$

where *ε*_*ρ*,12_ is the constant of proportionality, *χ*_s0_ = 10^−8^ m^3^·kg^−1^ and *ρ*_0_ = 1000 kg·m^−3^ are reducing constants. The value of *ε*_*ρ*,12_ can be determined by measurements of synthetic air, as described by Kleinrahm et al. (2019). The present experimental values are *ε*_vac,12_ = (− 57 ± 8) × 10^−6^ and *ε*_*ρ*,12_ = (66 ± 6) × 10^−6^ for the density sinker used in the top position of our improved measurement system. Please note that these two values depend on the mass of the sinker, and they will be different for other instruments. Values for the specific magnetic susceptibility for several fluids are given in our previous paper, e.g., *χ*_s_/*χ*_s0_ = − 0.61 for carbon dioxide. Rearranging Eqs. (1), (2), (3) and (4) yields the equation to calculate the fluid density:

(5) $$\rho_{\text{fluid}\;}=\frac{m_{\text{S},\text{vac}\;}^{*}-m_{\text{S},\text{fluid}\;}^{*}}{V_{\text{S}}}\cdot {\left(1+\varepsilon_{\text{vac},12}+\varepsilon_{\rho ,12}\cdot \frac{\chi_{\text{S}}}{\chi_{\text{S}0}}\cdot \frac{\rho_{\text{fluid}\;}-\rho_{\text{S}}}{\rho_{0}}\right)}^{-1}\text{,}$$

where m*_S,fluid_ = (*W*_2_ – *W*_1_)_fluid_/*α*, and *ρ*_S_ = *m*_S_/*V*_S_ is the density of the sinker. A detailed derivation of Eq. (5) is given in our previous paper (Kleinrahm et al. 2019); the last term in the parentheses in Eq. (5) was defined there as the “fluid-specific effect” *ε*_fse_. Please note that the volume of the sinker *V*_S_ depends on temperature and pressure. If the FTE were not taken into account, m*_S,vac_ would be replaced by the actual calibrated mass of the sinker *m*_S_, and this would cause a typical error of 0.23 kg·m^−3^ (Kleinrahm et al. 2019). Furthermore, if the terms in the parentheses in Eq. (5) were omitted, this would cause a typical error of 550 × 10^−6^·*ρ*_fluid_ (Kleinrahm et al. 2019).

### 3.2 Uncertainty of the fluid density

The uncertainty in density calculated with Eq. (5) was presented in our previous work (Kleinrahm et al. 2019). For a gravimetric sorption analyzer, density and adsorption are measured simultaneously while the latter one is the target. In this context, density can be either measured with the sorption analyzer and calculated with Eq. (5), or alternatively it could be calculated with a reference EOS using the measured temperature and pressure (and the analyzed composition in case of gas mixtures). In many cases, the densities of pure gases calculated with an EOS will yield a lower uncertainty. However, when gas mixtures are under investigation, Eq. (5) is recommended because even the state-of-the-art reference EOS for most gas mixtures cannot ensure a relative uncertainty better than 0.1%. For pure fluids for which reliable reference EOS exist (e.g., the equation of Span and Wagner from 1996 for CO_2_ has a relative uncertainty in density ranging from 0.03% to 0.05% at pressures up to 30 MPa and temperatures up to 523 K) and when the measured temperature and pressure are accurate enough (e.g., with an uncertainty in the order of 50 mK and 1.0 kPa, respectively, or less), the gas densities can be calculated with the reference EOS. In the present work, pure carbon dioxide was investigated, and the uncertainties in temperature and pressure measurements were low (see Sect. 2.2), and therefore, the densities were calculated with the reference EOS (Span and Wagner 1996). The combined expanded uncertainty in density *u*_C_(*ρ*) including the uncertainties in temperature and pressure was then calculated by

(6) $$u_{\text{C}}(\rho )={\left[u_{\text{EOS}}{\left(\rho \right)}^{2}+{\left({\left(\frac{\partial \rho }{\partial T}\right)}_{p}\cdot u(T)\right)}^{2}+{\left({\left(\frac{\partial \rho }{\partial p}\right)}_{T}\cdot u(p)\right)}^{2}\right]}^{0.5},$$

where *u*_EOS_(*ρ*) is the uncertainty in density of the reference EOS, and the partial derivatives were calculated with the reference EOS as well.

### 3.3 Determination of the adsorption on porous and non-porous material

In order to determine the absolute amount of adsorbed mass *m*_sorp_ on the adsorbent, Eq. (1) has to be extended to:

(7) $${\left(W_{1}-W_{0}\right)}_{\text{fluid}\;}=\left[m_{\text{CP}}-\rho_{\text{fluid}\;}\cdot \left(V_{\text{CP}}+V_{\text{sorp}\;}\right)+m_{\text{sorp}\;}\right]\cdot \alpha \cdot \phi_{01},$$

where *W*_0_ and *W*_1_ are the balance readings at the positions ZP and MP1, respectively, and *m*_sorp_ and *V*_sorp_ are the mass and volume of the adsorbed fluid. For a porous material, *m*_CP_ = (*m*_C_ + *m*_P_) and *V*_CP_ = (*V*_C_ + *V*_P_), where *m*_C_ and *V*_C_ are the mass and the volume of the container together with its lifting rod, and *m*_P_ and *V*_P_ are the mass and the volume of the porous sample inside the container. For a non-porous material, e.g., a sorption sinker instead of the container (see Fig. 3), *m*_CP_ and *V*_CP_ are the mass *m*_S_ and the volume *V*_S_ of the sorption sinker. The value *ϕ*_01_ is the coupling factor, which accounts for the FTE between the positions ZP and MP1; it was determined in the same way as the value *ϕ*_12_ using analogously Eqs. (2) to (4), but with the mass *m*_CP_ of the adsorbent instead of the mass of the sinker *m*_S_. For our improved measurement system, the values of *ε*_vac,01_ and *ε*_fluid,01_ are (− 34 ± 8) × 10^−6^ and (3 ± 1) × 10^−6^, respectively. With *V*_sorp_ = *m*_sorp_/*ρ*_sorp_, where *ρ*_sorp_ corresponds to the density of the adsorbed fluid, Eq. (7) can be rearranged to:

(8) $$m_{\text{sorp}\;}=\left[\frac{m_{\text{CP},\;\text{fluid}\;}^{*}}{\phi_{01}}-m_{\text{CP}}+\rho_{\text{fluid}\;}V_{\text{CP}}\right]\cdot {\left(\frac{\rho_{\text{sorp}\;}-\rho_{\text{fluid}\;}}{\rho_{\text{sorp}\;}}\right)}^{-1},$$

with m*_CP,fluid_ = (*W*_1_ – *W*_0_)_fluid_/*α*. Since the mass of the porous sample *m*_P_ was difficult to determine using a similar mass calibration technique as that for the solid sinkers, a measurement was carried out in an evacuated measurement cell after the activation of the porous sample. The value of *m*_P_ was then calculated by

(9) $$m_{\text{P}}=m_{\text{CP}}-m_{\text{C}}=\frac{m_{\text{CP},\text{vac}}^{*}}{\left(\varepsilon_{\text{vac},01}+1\right)}-m_{\text{C}},$$

where m*_CP,vac_ = (*W*_1_ – *W*_0_)_vac_/*α*. The coupling factor *ε*_vac,01_ was calculated analogously to Eq. (3), where the porous sample inside the container was replaced by a non-porous sample of a similar mass *m*_P_. Combining Eqs. (8) and (9) yields the result:

(10) $$m_{\text{sorp}\;}=\left[\frac{m_{\text{CP},\;\text{fluid}\;}^{*}}{1+\varepsilon_{\text{vac},01}+\varepsilon_{\text{fluid},01}}-\frac{m_{\text{CP},\text{vac}}^{*}}{1+\varepsilon_{\text{vac},01}}+\rho_{\text{fluid}\;}V_{\text{CP}}\right]\;\;\cdot {\left(\frac{\rho_{\text{sorp}\;}-\rho_{\text{fluid}\;}}{\rho_{\text{sorp}\;}}\right)}^{-1}$$

Equation (10) can also be used for the determination of the adsorbed mass on non-porous material (e.g., a sorption sinker). The influence of the FTE on the adsorbed mass *m*_sorp_ is taken into account by the terms *ε*_vac,01_ and *ε*_fluid,01_. If the FTE were not taken into account, m*_CP,vac_ would be replaced by the actual calibrated mass of the adsorbent *m*_CP_ (this would cause an error of *m*_CP_·*ε*_vac,01_), and the terms *ε*_vac,01_ and *ε*_fluid,01_ would be equal to zero in Eq. (10). It is important to note that, although the excess amount of adsorbed mass (*m*^ex^ = *m*_sorp_–*ρ*_fluid_·*V*_sorp_ by definition) does not require the term [(*ρ*_sorp_–*ρ*_fluid_)/*ρ*_sorp_]^−1^ and is an accepted thermodynamic quantity to report gas adsorption (Nguyen et al. 2018), the absolute one, as calculated by Eqs. (8) and (10), is presented in this work. The main reasons are: (1) most of the commonly used physically-based models for adsorption isotherms [e.g., Langmuir, Toth (1971) and Sips (1948) models] calculate the absolute adsorption; (2) it would be more useful to present the most comprehensive uncertainly analysis here, which can only be done by the analysis of the absolute adsorption and which can be easily simplified for the analysis of the excess adsorption.

The adsorption capacity of a porous material *q*_P_ and of a non-porous material *q*_NP_ can now be expressed by

(11) $$q_{\text{P}}=\left(m_{\text{sorp}}/M_{\text{filid}}\right)/m_{\text{P}},$$

and

(12) $$q_{\text{NP}}=\left(m_{\text{sorp}}/M_{\text{fluid}}\right)/A_{\text{NP}},$$

respectively, where *M*_fluid_ is the molar mass of the investigated fluid and *A*_NP_ is the estimated geometrical surface area of the sorption sinker (i.e., the area calculated by the overall geometry of the sinker and not including the effects of surface roughness). For porous materials, the adsorbed mass on the container surfaces can usually be neglected because its share is very small in comparison to the adsorbed mass on the porous sample.

### 3.4 Uncertainty of the adsorption

The combined uncertainty of the adsorbed mass can be determined according to the “Guide to the Expression of Uncertainty in Measurement” (ISO/IEC Guide 98–3 2008; GUM:1995) by applying the error propagation to Eq. (10) and simplifying it to:

(13) $$u_{\text{C}}{\left(m_{\text{sorp}}\right)}^{2}=\left\{u{\left(m_{\text{CP},\text{fluid}}^{*}\right)}^{2}+u{\left(m_{\text{CP},\text{vac}}^{*}\right)}^{2}+{\left[V_{\text{CP}}\cdot u_{\text{C}}\left(\rho_{\text{fluid}}\right)\right]}^{2}+{\left[\rho_{\text{fluid}\;}\cdot u\left(V_{\text{CP}}\right)\right]}^{2}\right\}\;\;\cdot {\left(1-\frac{\rho_{\text{fluid}}}{\rho_{\text{sorp}}}\right)}^{-2}+\frac{{\left(m_{\text{sorp}\;}\right)}^{2}}{\left(\rho_{\text{sorp}\;}-\rho_{\text{fluid}}\right)}\cdot {\left[u\left(\rho_{\text{fluid}}\right)+\frac{\rho_{\text{fluid}\;}}{\rho_{\text{sorp}\;}}\cdot u\left(\rho_{\text{sorp}}\right)\right]}^{2}$$

The major simplifications were the omission of the terms and the uncertainties of the apparatus contribution of the FTE *ε*_vac_ and the fluid contribution of the FTE *ε*_fluid_. The value of *ε*_vac_ has an absolute uncertainty of approximately 8 × 10^−6^ (Kleinrahm et al. 2019) and the sensitivity coefficient (which is approximately *ρ*_fluid_·*V*_CP_) for *u*(*ε*_vac_) is in the order of 0.1 g (assuming a gas density *ρ*_fluid_ of 100 kg·m^−3^ and a sample volume *V*_CP_ of 1.0 cm^3^); therefore, the uncertainty contribution of *ε*_vac_ to *u*_C_(*m*_sorp_) is less than 1 μg. The value of *ε*_fluid_ is in the order of 3 × 10^−6^ and its relative uncertainty is 10% (Kleinrahm et al. 2019). The sensitivity coefficient for *u*(*ε*_fluid_) is less than 10 g (assuming the mass of the adsorbent *m*_CP_ < 10 g); therefore, the uncertainty contribution of *ε*_fluid_ to *u*_C_(*m*_sorp_) is less than 3 μg. Please note that, although the influence of the terms and the uncertainties of *ε*_vac_ and *ε*_fluid_ to the combined uncertainty of the adsorbed mass *u*_C_(*m*_sorp_) is negligibly small, it does not mean that the influence of the FTE to the value of the adsorbed mass *m*_sorp_ can be neglected in all cases (see further discussions in Sects. 4.2 and 5.2).

By applying the error propagation to Eq. (11), the relative combined uncertainty of the adsorption capacity of a porous material *u*_C_(*q*_P_)/*q*_P_ can be calculated by

(14) $${\left[\frac{u_{\text{C}}\left(q_{\text{P}}\right)}{q_{\text{P}}}\right]}^{2}={\left[\frac{u_{\text{C}}\left(m_{\text{sorp}}\right)}{m_{\text{sorp}\;}}\right]}^{2}+{\left[\frac{u\left(m_{\text{P}}\right)}{m_{\text{P}}}\right]}^{2},$$

where the uncertainty of the molar mass was negligibly small, and the term *u*(*ρ*_fluid_)/*ρ*_sorp_ in Eq. (13) can be neglected because it is in the order of a few 10^−5^. At low fluid densities, Eq. (14) underestimates the uncertainty in *q*_p_ because uncertainties in the weighings place a lower limit on the absolute combined uncertainty of *q*_P_:

(15) $$u_{\text{C},\min}{\left(q_{\text{P}}\right)}^{2}=\frac{u{\left(m_{\text{CP},\;\text{fuid}}^{*}\right)}^{2}+u{\left(m_{\text{CP},\text{vac}}^{*}\right)}^{2}}{{\left(M_{\text{fluid}\;}\cdot m_{\text{P}}\right)}^{2}},$$

When a non-porous material is investigated, the relative combined uncertainty of the adsorption capacity of a non-porous material *u*_C_(*q*_NP_)/*q*_NP_ can be calculated by applying the error propagation to Eq. (12) and simplifying to:

(16) $${\left[u_{\text{C}}\left(q_{\text{NP}}\right)\right]}^{2}={\left[\frac{u_{\text{C}}\left(m_{\text{sorp}}\right)}{m_{\text{sorp}\;}}\right]}^{2}\cdot q_{\text{NP}}^{2},$$

where the uncertainty of the molar mass was negligibly small. The term *u*(*A*_NP_)/*A*_NP_, estimated to be 1.0%, was neglected as well, because it was relatively small compared to the term *u*_C_(*m*_sorp_)/*m*_sorp_, which was generally larger than 10.0%. The value *u*_C_(*m*_sorp_) in Eq. (16) was calculated according to Eq. (13). However, since the adsorbed mass *m*_sorp_ was less than 50 μg in the pressure range *p* < 0.99·*p*_s_, where *p*_s_ is the dew-point pressure, and in the order of 200 μg in the pressure range 0.99·*p*_s_ ≤ *p* < *p*_s_ (see Sect. 5.1), the contribution of the terms multiplied by (*m*_sorp_)^2^ in Eq. (13) to *u*_C_(*m*_sorp_) is less than 5 μg, which is much smaller than the first term and can therefore be neglected. The minimum absolute combined uncertainty in *q*_NP_ can be estimated by

(17) $$u_{\text{C},\min}{\left(q_{\text{NP}}\right)}^{2}=\frac{u{\left(m_{\text{CP},\text{fluid}}^{*}\right)}^{2}+u{\left(m_{\text{CP},\text{vac}}^{*}\right)}^{2}}{{\left(M_{\text{fluid}}\cdot A_{\text{NP}}\right)}^{2}},$$

for fluid densities *ρ*_fluid_ less than about 10 kg m^−3^.

## 4 Sorption measurement on porous materials

### 4.1 Measurements and results

Test measurements of the adsorption of carbon dioxide on zeolite 13X were conducted with our improved measurement system used as a gravimetric sorption analyzer. Measurements were carried out along the isotherm *T* = 283.144 K with pressure-increasing steps from *p* = 0.0001 MPa up to the dew-point pressure and then with pressure-decreasing steps. Information of the carbon dioxide sample is summarized in Table 2; it was used as received from the supplier without further gas analysis or purification. The zeolite 13X sample (Chemiewerk Bad Köstritz GmbH, Germany, type: Köstrolith 13XBFK, surface *A* = 500 m^2^·g^−1^) was provided in binder-free ball granules (diameter of approximately 2 mm) and was used as received. Before an isothermal measurement, the zeolite sample was activated inside the measuring cell at a temperature *T* = 523 K and a pressure less than 0.1 Pa for at least 4 h (overnight for the very first time of activation after the sample was put into the measuring cell). The mass of the zeolite sample *m*_P_ was obtained after activation by weighing it in the evacuated measurement cell using Eq. (9); the volume of the sample *V*_P_ was determined by measuring the buoyancy force on the sample in helium at *T* = 293.15 K and *p* at (2.0, 4.0, 6.0 and 8.0) MPa, with the assumption that helium is not adsorbed on zeolite. Although it has been shown that this assumption may be problematic (Maggs et al. 1960; Malbrunot et al. 1997; Hocker et al. 2003; Pini 2014; and Brandani et al. 2016), this volume determination method is by far the most commonly used and the most reliable one available to us. Besides, we estimated the relative uncertainty of *V*_P_ to be as large as 2.0% (see Sect. 4.2), compared to which, the uncertainty of *V*_P_ attributed to this assumption (hard to quantify) should be negligibly small, otherwise obvious helium adsorption would be observed. Information for the helium sample is summarized in Table 2; the density of helium was calculated with a reference EOS (Ortiz-Vega 2013). The mass and volume of the zeolite sample were *m*_P_ = (2.1549 ± 0.0431) g and *V*_P_ = (0.3415 ± 0.0068) cm^3^; the second value within the parentheses is the expanded uncertainty (*k* = 2). The sample container for the zeolite sample was a hollow cylinder (outer diameter: 16 mm, wall thickness: 0.5 mm, height: 20 mm, material: stainless steel) with a bottom plate and a hanger on top. The mass and the volume of the container were *m*_C_ = (2.4093 ± 0.0004) g and *V*_C_ = (0.30466 ± 0.00035) cm^3^ using typical mass and volume calibration methods for solids.

**Fig. 4:**
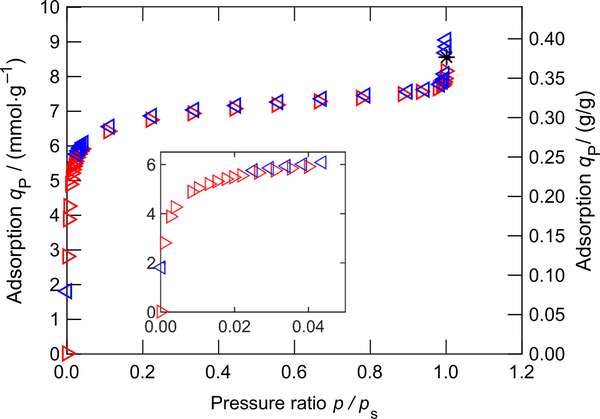
Adsorption capacity *q*_P_ of zeolite 13X for carbon dioxide along the isotherm *T* = 283.144 K. The expanded combined uncertainties are within the vertical size of the plotted symbols. The measurements were conducted with increasing (⊳) and decreasing pressures (⊲). The turning point (*) was in the gas–liquid coexistence region; the measured pressure *p* = 4.5088 MPa was higher than the dew-point pressure *p*_s_ = 4.5033 MPa, calculated with the reference equation of Span and Wagner (1996), at the measured temperature *T* = 283.160 K

The measurement results are listed in Table 3 and illustrated in Fig. 4. It can be seen that the adsorption shows a typical type I curve (Brunauer et al. 1940), which is the wellknown Langmuir adsorption isotherm, i.e., the adsorbed mass increases significantly at low pressure (*p*/*p*_s_ < 0.05) and then reaches a plateau until close to the dew-point pressure. When the dew-point pressure was approached, the adsorption capacity increased again, implying the effect of the capillary condensation. The results are in good agreement with literature (e.g., Cavenati et al. 2004).

### 4.2 Uncertainty analysis

As has been discussed in Sect. 2.2, for our improved measurement system, the expanded uncertainty (*k* = 2) in temperature and pressure measurements are 16 mK and (0.2 to 0.7) kPa, respectively. The relative expanded uncertainty (*k* = 2) in density calculated with equation of Span and Wagner (1996) over the investigated (*p*,*T*) range was 0.03%. The combined uncertainty in the density of the sample fluid was calculated by Eq. (6). The expanded uncertainty (*k* = 2) of m*_CP,vac_ and m*_CP,fluid_ was estimated to be 60 μg according to the fluctuation of the weighing values. The major contribution to the uncertainty (*k* = 2) of the volume of the adsorbent *V*_CP_ was the uncertainty of the volume of the zeolite sample *V*_P_, which was estimated by

(18) $$u{\left(V_{\text{P}}\right)}^{2}=\frac{u{\left(m_{\text{CP},\;\text{fluid}}^{*}\right)}^{2}+u{\left(m_{\text{CP},\text{vac}}^{*}\right)}^{2}}{\rho_{\text{He},293.15\;\text{K}}^{2}},$$

and thus *u*(*V*_CP_) = *u*(*V*_P_) = 0.0034 cm^3^. The standard uncertainty of the mass of the zeolite sample was estimated to be *u*(*m*_P_) = 0.02155 g, corresponding to a sample purity of 99.0 mass-%. (In other words, the impurities in the zeolite sample, which amount to 1% of (*m*_P_ = 2.1549 g), are assumed not to adsorb gases).

The value of *ρ*_sorp_ is commonly estimated as the saturated-liquid density at the standard boiling point pressure 0.1 MPa (e.g., Dreisbach et al. 1999; Cavenati et al. 2004). However, since the triple-point pressure of carbon dioxide *p*_tr_ = 0.518 MPa at *T*_tr_ = 216.6 K is greater than 0.1 MPa, the value of *ρ*_sorp_ was estimated to be the saturated-liquid density at the pressure *p*_tr_, i.e., *ρ*_sorp_ = 1178 kg m^−3^ as calculated with the reference EOS for carbon dioxide (Span and Wagner 1996). For a porous material as an adsorbent, we assume that the true density *ρ*_sorp_ of the adsorbed sample fluid (which was carbon dioxide in the present case) on the surface of the porous zeolite can be considerably larger than the saturated-liquid density at the triple-point temperature of carbon dioxide, especially for the first molecular layer on the surface. We estimate that this assumption involves a relative expanded uncertainty (*k* = 2) of 10%, which could, however, be significantly larger.

**Fig. 5:**
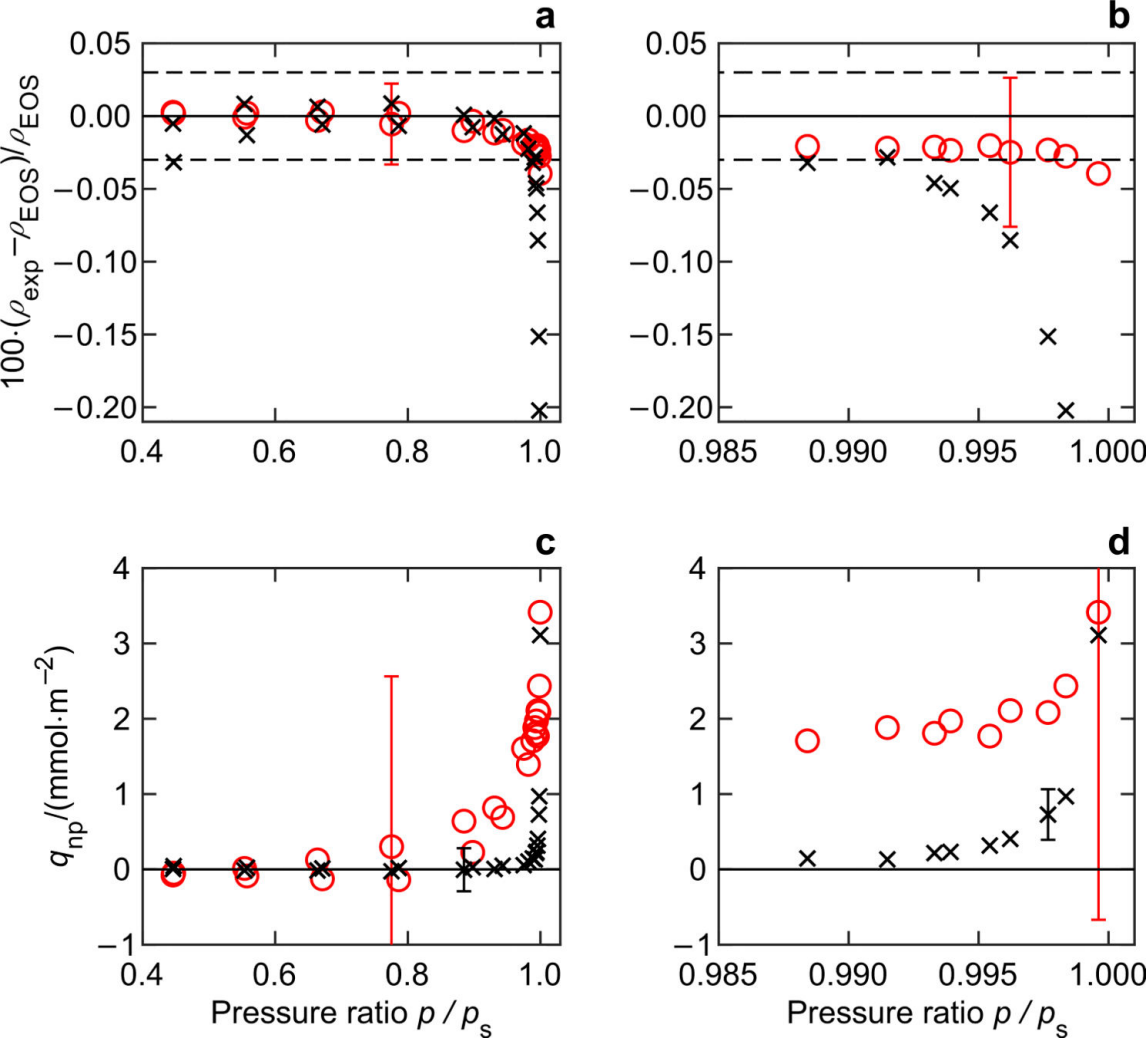
**a**, **b** Relative deviations of the experimental densities *ρ*_exp_ for carbon dioxide at *T* = 283.175 K from densities *ρ*_EOS_ calculated with reference equation of state (Span and Wagner 1996). **c**, **d** Adsorption *q*_NP_ of carbon dioxide on the surface of the two solid sinkers. The saturated pressure of carbon dioxide at *T* = 283.175 K is *p*_s_ = 4.5050 MPa. ○, measured with the density sinker; ×, measured with the sorption sinker (see Table 1). The dashed lines in panels (**a**) and (**b**) are the uncertainty boundaries (*k* = 2) of the reference equation. An error bar (expanded combined uncertainty *k* = 2) for one measurement is plotted in each figure

For a non-porous material as an adsorbent (e.g., our sorption sinker, see Fig. 3), we assume that the first molecular layer on its surface has the same density as on the porous zeolite. In the vicinity of the dew-point pressure, however, capillary condensation can occur, and a thin liquid film covers the surface; its (hypothetical) thickness can be in the order of up to about 0.1 μm (see Fig. 5c). We estimate the density *ρ*_sorp_ of this liquid film (including the first adsorbed molecular layer) as the saturated-liquid density of carbon dioxide at the measured temperature *T* = 283.150 K to be *ρ*_sorp_ = *ρ*_sat,liq_ = 861 kg m^−3^ [calculated with the reference EOS of Span and Wagner (1996)]. Furthermore, we estimate that this assumption *ρ*_sorp_ = *ρ*_sat,liq_ involves a relative expanded uncertainty (*k* = 2) of 10%, however, it could be much greater.

Since the surface area of zeolite *A*_NP_ is in the order of 500 m^2^·g^−1^, while the surface area of the sample container was approximately 24.0 cm^2^, the adsorption on the walls of the sample container was ignored. The influence of this simplification on the resulting adsorption capacity is negligibly small (less than 3 × 10^−6^). If the FTE was not taken into consideration, see Eq. (10) and the following comments, with *m*_sorp_ (= 700 mg) the error would be on the order of 0.2 mg for the current case, which corresponds to approximately 0.002 mmol·g^−1^ or 0.03% in adsorption capacity *q*_P_ for a typical (*p*,*T*) state, and this was negligibly small. Hence, in case of sorption measurements of gases (except for hydrogen and helium) on porous materials, the influence of the FTE on the determination of the adsorbed mass *m*_sorp_ is negligibly small. Recent helium adsorption measurements on clinoptilolite carried out by Arami-Niya et al. (2019) using a gravimetric sorption analyzer obtained an adsorption capacity around 0.15 mmol·g^−1^ or less at pressures below 5 MPa. In this case, the influence of the FTE may be non-negligible.

The relative combined expanded uncertainty (*k* = 2) of the adsorption capacity for each measuring point is listed in Table 3. The uncertainty budget for the adsorption capacity *q*_P_ of zeolite 13X for carbon dioxide, measured with our improved measurement system, is summarized in Table 4; the measurement at *T* = 283.165 K and *p* = 3.9881 MPa was taken as an example. As can be seen in Table 4, the uncertainty of the mass of the zeolite sample *u*(*m*_P_) is the dominating one, followed by that of the density of the adsorbed fluid *u*(*ρ*_sorp_). The contribution of the volume of the adsorbent *u*(*V*_CP_) to the uncertainty is one order lower than the previous two parameters, and those of the remaining parameters (temperature, pressure, fluid density in the gas phase, and weighing values) are negligibly small. Please note that, if the excess adsorption was presented rather than the absolute one, the second dominating uncertainty *u*(*ρ*_sorp_) can be ignored in the uncertainty calculation, i.e., the uncertainty of the measured excess adsorption is lower than that of the absolute one for porous materials. In summary, the uncertainty of the adsorption capacity of a porous material is largely attributed to the porous sample itself rather than to the measurement technique of the gravimetric sorption analyzer; this agrees with various studies (Hocker et al. 2003; Pini 2014; and Brandani et al. 2016), which concluded that the estimation of the mass and/or volume of the adsorbent involves a non-negligible uncertainty contribution to adsorption meausurements.

In Table 4, the uncertainty budget of a typical commercial gravimetric sorption analyzer is also listed. The expanded uncertainties (*k* = 2) in the measurements of temperature, pressure, weighing values m*_CP,vac_ and m*_CP,fluid_ were estimated to be 300 mK, 3.5 kPa, 80 μg, and 80 μg, respectively. The uncertainty of the volume of the adsorbent would then change to 0.0090 cm^3^ according to Eq. (18). As seen in Table 4, the combined uncertainty in the adsorption capacity of zeolite 13X for carbon dioxide measured with a typical commercial gravimetric sorption analyzer is similar to that measured with our the improved measurement system; i.e., the improvement of our measurement system did not significantly reduce the uncertainty in the sorption measurement of carbon dioxide on zeolite 13X. However, if an adsorption system (gas on adsorbent) with much lower adsorption (e.g., methane on shale, or hydrogen on metal–organic frameworks) were under investigation, the improvement could be beneficial. Furthermore, for the purpose of accurate determination of the adsorption on non-porous material (e.g., the solid sinker with rough surface in this work), the improvement was significant as discussed in the next section.

## 5 Sorption measurements on a solid sinker

### 5.1 Measurement and results

Test measurements of the adsorption of carbon dioxide on the surface of the sorption sinker was conducted along the isotherm *T* = 283.175 K, using the improved measurement system as a tandem-sinker densimeter. Measurements were carried out with pressure-increasing steps from *p* = 2 MPa up to the dew-point pressure and then with pressure-decreasing steps.

Assuming that there is no adsorption on both the density sinker and the sorption sinker, the measured densities using both sinkers are listed in Table 5. When the density was calculated using the sorption sinker, an equation analogous to Eq. (5) was used with m*_S,vac_ replaced by m*_CP,vac_, m*_S,fluid_ replaced by m*_CP,fluid_, and furthermore, with the volume *V*_S_ and the density *ρ*_S_ of the sorption sinker, and the values *ε*_vac,01_ and *ε*_*ρ*,01_ for the change in height of the permanent magnet between the positions ZP and MP1. Relative deviations of the experimental densities of carbon dioxide from values calculated with the reference EOS (Span and Wagner 1996) are illustrated in Fig. 5a, b. As shown in these figures, the measured densities agree with the reference EOS within mutual uncertainties at pressures lower than 0.85·*p*_s_. However, when the dew point is approached, the measured densities, especially those calculated with the sorption sinker, are distorted, which implies a significant impact from surface phenomena (e.g., adsorption and capillary condensation). The trend of the deviation of the experimental values in Fig. 5a, b for the sorption sinker qualitatively agree with the theoretical calculation results of Philip (1978), in analyzing combined adsorption and capillary condensation on rough surfaces (see Fig. 8 in Philip 1978), and with the results of Herminghaus (2012), in calculating the adsorption isotherms on surfaces with Gaussian roughness (see Fig. 3 in Herminghaus 2012).

When adsorption (and capillary condensation) of the sample gas on the sinker surface was taken into consideration, the adsorption capacity *q*_NP_ (see Eq. (12)) of both sinkers for carbon dioxide are listed in Table 5 and illustrated in Fig. 5c, d; please note that the density of carbon dioxide was calculated with a reference EOS (Span and Wagner 1996). When the adsorbed mass on the surface of the density sinker was calculated, an equation analogous to Eq. (10) was used with m*_CP,fluid_ replaced by m*_S,fluid_, m*_CP,vac_ replaced by m*_S,vac_, and furthermore, with the volume *V*_S_ and the density *ρ*_S_ of the density sinker, and the values *ε*_vac,12_ and *ε*_fluid,12_ for the change in height of the permanent magnet between the positions MP1 and MP2. As can be seen in Fig. 5c, d and listed in Table 5, the adsorption capacity *q*_NP_ of the density sinker at all measuring points are not reliable because the value is smaller than its uncertainty (see Sect. 5.2). In contrast to that, the adsorption capacity *q*_NP_ on the surface of the sorption sinker in the vicinity of the dew point (*p* > 0.995·*p*_s_) is reliable (the value is larger than its uncertainty) with values up to *q*_NP_ = 3.0 mmol·m^−2^. This result implies that in the vicinity of the dew point (*p* > 0.995·*p*_s_), capillary condensation dominates the surface interaction between the sample fluid and the solid surfaces, and the adsorption capacity can be measured by our improved measurement system. Further studies on surface phenomena in the vicinity of the dew point of pure fluids and fluid mixtures, using sinkers with different surface characteristics (e.g., with gold plated surfaces), are presented by Yang and Richter (2020).

### 5.2 Uncertainty analysis

For our improved measurement system, the combined uncertainty in density of the sample fluid was calculated by Eq. (6), with the expanded uncertainties (*k* = 2) in temperature, pressure and density being 16 mK, (0.2 to 0.7) kPa and 0.03% (Span and Wagner 1996), respectively. The expanded uncertainty (*k* = 2) of the weighing values m*_CP,fluid_ and m*_CP,vac_ were both estimated to be 60 μg. The relative expanded uncertainties (*k* = 2) of the volume of the sinkers were obtained from the volume calibration (see Table 1), i.e., *U*(*V*_S_)*/V*_S_ = 0.001% for the density sinker and *U*(*V*_CP_)*/V*_CP_ = 0.02% for the sorption sinker. The combined uncertainty in sorption capacity *q*_NP_ was then calculated with Eq. (16). If the FTE was not taken into consideration, see Eq. (10) and the following comments, the error of *m*_sorp_ would be in the order of 0.3 mg for the current case, which corresponds to 0.7 mmol·m^−2^ or about 22% of the adsorption capacity *q*_NP_ for the (*p*,*T*) state with the highest adsorption, i.e., for the present measurements, near the dew-point pressure (see Table 5). Hence, for a non-porous material, the influence of the FTE has to be taken into account.

The uncertainty budget for the adsorption capacity *q*_NP_ of both sinkers for carbon dioxide, measured with our improved measurement system, is summarized in Table 6, with the measurement at *T* = 283.208 K and *p* = 3.9857 MPa as an example. Each of the uncertainty parameters (measurements of temperature, pressure and weighing values; density calculated with reference EOS; volume of the sinkers), which are presented in Table 6, has a non-negligible influence to the combined uncertainty of the adsorption capacity. Since the uncertainty of the density of the adsorbed fluid *u*(*ρ*_sorp_) is not a dominant factor, the excess adsorption and the absolute adsorption for nonporous materials are almost at the same level of uncertainty. As can be seen in Table 6, the uncertainty in adsorption capacity *q*_NP_ of the density sinker is much larger than that of the sorption sinker. The main reason is that the surface-to-volume ratio of the sorption sinker is approximately 18.2 times greater than that of the density sinker. Therefore, the contribution of the combined uncertainty in density for the density sinker was approximately 18.2 times greater than that for the sorption sinker, as can be derived from Eqs. (12) and (16). The combined expanded uncertainty (*k* = 2) of the adsorption capacity *q*_NP_ for each measuring point are listed in Table 5. The values of the adsorption capacity of the density sinker at all measuring points are smaller than their expanded uncertainty, while the values of the adsorption capacity of the sorption sinker are larger in the vicinity of the dew point (*p* > 0.995·*p*_s_). Therefore, our improved measurement system was accurate enough for the investigation of the adsorption on the sorption sinker, but not for the density sinker.

In Table 6, the uncertainty budget of a typical commercial gravimetric sorption analyzer is also listed. As in Sect. 4.2, the expanded uncertainties (*k* = 2) in the measurements of temperature, pressure, and weighing values were estimated to be 300 mK, 3.5 kPa, and 80 μg, respectively. The relative expanded uncertainties (*k* = 2) of the volume of the density sinker were obtained from a volume calibration certificate provided by the manufacturer (Rubotherm), i.e., *U*(*V*_S_)*/V*_S_ = 0.05% and we assume the same uncertainty for the sorption sinker *U*(*V*_CP_)*/V*_CP_ = 0.05%. As can be seen in Table 6, the uncertainty in the adsorption capacity *U*_C_(*q*_NP_) of the sinkers using a typical commercial gravimetric sorption analyzer is much larger than that of our improved measurement system. The uncertainty is larger than the sorption capacity *q*_NP_ itself at all measuring points for both sinkers. Therefore, a typical commercial gravimetric sorption analyzer is not accurate enough to measure the adsorption on the surface of a quasi non-porous material significantly better than its uncertainty.

## 6 Conclusion

For the determination of the adsorption of gases on porous and quasi non-porous materials, the technique of a commercial gravimetric sorption analyzer was investigated, and a detailed uncertainty analysis was presented. The uncertainty analysis was applied to both a typical commercial apparatus and a measurement system improved by us. The force-transmission error (FTE) of the magnetic-suspension coupling was also taken into account.

As a representative of porous material, Zeolite 13X was used, and the test measurements were conducted with carbon dioxide along the isotherm *T* = 283.144 K from *p* = 0.0001 MPa up to the dew-point pressure. The measurement results agree with reliable literature data. The uncertainty of the adsorption capacity of the porous material is largely attributed to the porous sample itself (mass and volume of the sample) rather than the measurement technique with a gravimetric sorption analyzer. The influence of the FTE on the uncertainty of the adsorbed mass on porous material was negligibly small.

Adsorption measurements were also carried out with carbon dioxide on solid sinkers (quasi non-porous material) along the isotherm *T* = 283.175 K from *p* = 2 MPa up to the dew-point pressure. Two sinkers were investigated: a density sinker with a relatively small surface-to-volume ratio and a smooth surface, and a sorption sinker with a relatively large surface-to-volume ratio and a rough surface. The most important uncertainty contributions in the adsorption measurement on a non-porous material are the weighing values of the balance, the density of the investigated fluid in the gas phase, and the volume of the non-porous material. The uncertainty analysis demonstrated that our improved measurement system was able to measure the condensed mass of the sample gas on the surface of the sorption sinker in the vicinity of the dew point (capillary condensation), while the accuracy of a typical commercial apparatus was clearly not sufficient. In case of a quasi non-porous material, the influence of the FTE had a significant impact.

## Figures and Tables

**Table 1:** Specification of the sinkers used

Sinker	Material	*m*/g	10^6^ *U*(*m*)/*m*	*V*_0_/cm^3^	10^6^ *U*(*V*_0_)/*V*_0_	*A*/cm^2^	*U*(*A*)/*A*
Ti20^a^	Titanium	19.65711	20	4.360347	10	18.1	0.02
SS09^b^	SS304^c^	9.33899	60	1.17931	200	89.2	0.02

The mass and volume of the Ti20 sinker (density sinker) were determined at NIST, while the mass and volume of the sinker SS09 (sorption sinker) were determined at Ruhr University Bochum. *U*(*m*)/*m*, *U*(*V*_0_)/*V*_0_ and *U*(*A*)/*A* are the relative expanded uncertainty (*k* = 2) of the mass, volume and geometrical surface area, respectively. *V*_0_ is the volume of the sinker at reference state (*p*_0_ = 0.101325 MPa and *T*_0_ = 293.15 K)

a The density sinker is a cylinder with outer diameter *d*_o_ = 18.2 mm, concentric inner diameter *d*_i_ = 5.0 mm, and height *h* = 18.2 mm; surface polished with abrasive to obtain a smoother finish

b Sorption sinker with a base ring *d*_o_ = 20.0 mm, *d*_i_ = 5.0 mm, *h* = 6.0 mm, and three upper rings: thickness 0.1 mm, *d*_o_ = (11.0, 15.0, 19.0) mm, and *h* = 27.0 mm (see Fig. 3, bottom sinker). The base ring was made on a lathe, and the upper rings were made on a rolling machine. The surfaces of the upper rings were sandblasted with 250 μm particles

c Type 1.4301 stainless steel (equivalent to SAE/ANSI type 304)

**Table 2:** Sample information

Chemical name	Source	Purity/mole fraction	Purification method
Carbon dioxide	Air Products	0.999995^a^	None
Helium	Air Liquide	0.99999^b^	None

a Impurities (stated by supplier): *x*(H_2_O) ≤ 2.0 × 10^−6^, *x*(O_2_) ≤ 0.5 × 10^−6^, *x*(C_m_H_n_) ≤ 0.1 × 10^−6^, *x*(N_2_) ≤ 2.0 × 10^−6^, *x*(CO) ≤ 0.5 × 10^−6^, where *x* denotes mole fraction

b Impurities (stated by supplier): *x*(H_2_O) ≤ 2.0 × 10^−6^, *x*(O_2_) ≤ 2.0 × 10^−6^, *x*(C_m_H_n_) ≤ 0.2 × 10^−6^, (N_2_) ≤ 0.5 × 10^−6^

**Table 3:** Adsorption capacity *q*_P_ of zeolite 13X for carbon dioxide along the isotherm *T* = 283.144 K and the relative combined expanded uncertainty (*k* = 2) *U*_C_(*q*_P_)/*q*_P_, where *T* is the temperature (ITS-90) and *p* is the pressure

*T*/K	*p*/MPa	*q*_P_/(mmol g^−1^)	*U*_C_(*q*_P_)/*q*_P_ ·100	*T*/K	*p*/MPa	*q*_P_/(mmol g^−1^)	*U*_C_(*q*_P_)/*q*_P_ ·100
283.164	0.0001	0.0163	5.83	283.165	3.9881	7.4934	2.20
283.186	0.0042	2.8150	2.00	283.146	4.2039	7.5597	2.24
283.176	0.0118	3.8814	2.00	283.140	4.4040	7.6584	2.28
283.166	0.0176	4.2648	2.00	283.137	4.4444	7.7155	2.29
283.161	0.0379	4.8956	2.00	283.137	4.4572	7.7497	2.30
283.157	0.0458	5.0425	2.00	283.126	4.4667	7.7871	2.30
283.161	0.0590	5.2144	2.00	283.117	4.4770	7.8449	2.30
283.151	0.0684	5.3198	2.00	283.112	4.4864	7.9459	2.31
283.138	0.0796	5.4189	2.00	283.131	4.4968	8.1605	2.31
283.137	0.0887	5.4906	2.00	283.160	4.5088^a^	8.5585	8.04
283.130	0.0987	5.5595	2.00	283.156	4.5082^a^	8.8643	8.01
283.128	0.1191	5.6736	2.00	283.142	4.5044^a^	9.0577	7.99
283.136	0.1394	5.7660	2.00	283.129	4.4934	8.6850	2.31
283.139	0.1591	5.8446	2.00	283.136	4.4840	8.0751	2.30
283.147	0.1799	5.9139	2.00	283.136	4.4756	7.9181	2.30
283.155	0.4914	6.4251	2.00	283.132	4.4630	7.8587	2.30
283.166	0.9930	6.7524	2.01	283.121	4.4289	7.7750	2.29
283.164	1.4912	6.9385	2.02	283.116	4.2479	7.6249	2.25
283.173	1.9886	7.0729	2.03	283.114	4.0460	7.5639	2.21
283.176	2.4895	7.1842	2.06	283.107	2.5099	7.2659	2.06
283.171	2.9911	7.2843	2.09	283.096	2.0078	7.1640	2.03
283.168	3.4929	7.3825	2.14				

The expanded uncertainties (*k* = 2) of the measurements are 16 mK for temperature *T*, and between (0.1 and 0.7) kPa for pressure *p*. The needed densities *ρ*_fluid_ (see Eqs. (10) and (11)) were calculated with the reference equation of state (Span and Wagner 1996) with a relative expanded uncertainty (*k* = 2) of 0.03%

a State points in the gas–liquid coexistence region; the saturated pressure (e.g., *p*_s_ = 4.5033 MPa at *T*_s_ = 283.160 K) was estimated by the reference equation of state for carbon dioxide (Span and Wagner 1996)

**Table 4:** Uncertainty budget for the adsorption capacity *q*_P_ for carbon dioxide on zeolite 13X (see Eq. (14))

Source^a^	Uncertainty *U* (*k* = 2)	Contribution to *U*_C_(*q*_P_)/*q*_P_
*Our improved gravimetric sorption analyzer*		
Temperature *T*	16 mK	(0.0021%)
Pressure *p*	0.2 kPa	(0.0018%)
Density calculated with equation of Span and Wagner (1996)	0.03%	(0.0032%)
Combined uncertainty in density of the sample fluid *U*_C_(*ρ*)^b^	0.043 kg·m^−3^	0.0043%
Weighing value m*_CP,vac_ (4.5897 g)	60 μg	0.0093%
Weighing value m*_CP,fluid_ (5.0703 g)	60 μg	0.0093%
Volume of the adsorbent *U*(*V*_CP_) (*V*_CP_ ≈ 0.6462 cm^3^)	0.0068 cm^3^	0.1136%
Density of condensed fluid *U*(*ρ*_sorp_)/*ρ*_sorp_^c^	10%	0.9155%
Mass of zeolite sample *m*_P_ (2.1549 g)	0.0431 g	2.0000%
Relative combined expanded uncertainty (*k* = 2) in adsorption capacity^d^ *U*_C_(*q*_P_)/*q*_P_		2.2025%
*A typical commercial gravimetric sorption analyzer*	300 mK	(0.0401%)
Temperature *T*	300 mK	(0.0401%)
Pressure *p*	3.5 kPa	(0.0158%)
Density calculated with equation of Span and Wagner (1996)	0.03%	(0.0032%)
Combined uncertainty in density of the sample fluid *U*_C_(*ρ*)^b^	0.055 kg·m^−3^	0.0433%
Weighing value m*_CP,vac_ (4.5897 g)	80 μg	0.0124%
Weighing value m*_CP,fluid_ (5.0703 g)	80 μg	0.0124%
Volume of the adsorbent *U*(*V*_CP_) (*V*_CP_ ≈ 0.6462 cm^3^)	0.0090 cm^3^	0.1504%
Density of condensed fluid *U*(*ρ*_sorp_)*/ρ*_sorp_^c^	10%	0.9155%
Mass of zeolite sample *m*_P_ (2.1549 g)	0.0431 g	2.0001%
Relative combined expanded uncertainty (*k* = 2) in adsorption capacity^d^ *U*_C_(*q*_P_)/*q*_P_		2.2053%

As an example, the measurement at (*T* = 283.165 K, *p* = 3.9881 MPa, *ρ* = 107.851 kg·m^−3^) of carbon dioxide was taken (see Table 3)

Adsorbed mass on the zeolite sample *m*_sorp_ ≈ (0.7107 ± 0.0066 g); *m*_sorp_/*m*_P_ ≈ 0.3298 g/g

a The influence of the uncertainties of the FTE correction factors *ε*_vac_ and *ε*_fluid_ for the sample container with zeolite is negligibly small. Therefore, they are not listed in the table

b Combined uncertainty according to Eq. (6), which includes the uncertainty in temperature and pressure measurement, and the uncertainty of the density calcuated with the reference equation of Span and Wagner (1996) for carbon dioxide

c *ρ*_sorp_ ≈ 1178 kg·m^−3^. The assumption of the density *ρ*_sorp_ is explained in Sect. 4.2. Its uncertainty was estimated to be 10%, however, it could be much larger; see discussion in Sect. 4.2

d Calcuated with Eq. (14)

**Table 5:** Adsorption *q*_NP_ of carbon dioxide on the surface of the density sinker and the sorption sinker along the isotherm *T* = 283.175 K, where *T* is the temperature (ITS-90), *p* is the pressure, and *U*_C_(*q*_NP_) is the expanded combined uncertainty (*k* = 2) of *q*_NP_

			Sorption sinker				Density sinker			
*T*/K	*p*/MPa	*ρ*_EOS_/kg·m^−3^	*ρ*_exp_/kg·m^−3^	100·Δ*ρ*/*ρ*	*q*_NP_/mmol·m ^−2^	*U*_C_(*q*_NP_)/ mmol·m^−2^	*ρ*_exp_/kg·m^−3^	100·Δ*ρ*/*ρ*	*q*_NP_/mmol·m^−2^	*U*_C_(*q*_NP_)/mmol·m^−2^
283.178	2.0081	43.1937	43.1915	−0.005	0.008	0.232	43.1949	0.003	−0.075	1.463
283.196	2.4943	56.0370	56.0417	0.008	− 0.015	0.239	56.0365	− 0.001	0.013	1.645
283.204	2.9900	70.6669	70.6714	0.006	− 0.018	0.249	70.6648	− 0.003	0.126	1.897
283.206	3.4935	87.7322	87.7398	0.009	− 0.033	0.263	87.7274	− 0.005	0.301	2.263
283.208	3.9857	107.685	107.686	0.001	− 0.025	0.286	107.674	− 0.010	0.640	2.827
283.210	4.1936	117.603	117.601	− 0.002	− 0.028	0.300	117.589	− 0.012	0.816	3.192
283.207	4.3909	128.269	128.254	− 0.012	0.005	0.321	128.245	− 0.019	1.607	3.685
283.197	4.4528	131.965	131.923	− 0.032	0.084	0.329	131.937	− 0.021	1.707	3.890
283.190	4.4667	132.830	132.792	− 0.028	0.069	0.331	132.801	− 0.022	1.883	3.941
283.178	4.4776	133.538	133.472	− 0.050	0.166	0.333	133.507	− 0.024	1.971	3.984
283.180	4.4880	134.180	134.066	− 0.085	0.334	0.335	134.147	− 0.025	2.109	4.023
283.186	4.4976	134.775	134.502	− 0.202	0.899	0.337	134.737	− 0.028	2.435	4.060
283.183	4.5033^a^	135.141	134.266	− 0.648	3.039	0.343	135.087	− 0.039	3.415	4.084
283.182	4.4945	134.589	134.385	− 0.151	0.657	0.336	134.558	− 0.023	2.084	4.049
283.179	4.4844	133.960	133.871	− 0.066	0.250	0.334	133.933	− 0.020	1.770	4.010
283.178	4.4748	133.365	133.304	− 0.046	0.150	0.333	133.337	− 0.021	1.808	3.974
283.174	4.4231	130.233	130.203	− 0.023	0.048	0.325	130.211	− 0.016	1.393	3.793
283.161	4.2488	120.534	120.519	− 0.013	0.013	0.306	120.522	− 0.010	0.690	3.317
283.162	4.0450	110.467	110.458	− 0.008	0.003	0.290	110.463	− 0.004	0.226	2.923
283.150	3.5420	89.5769	89.5709	− 0.007	0.010	0.265	89.5786	0.002	− 0.138	2.308
283.153	3.0230	71.7367	71.7325	− 0.006	0.010	0.250	71.7387	0.003	− 0.126	1.918
283.156	2.5102	56.4965	56.4891	− 0.013	0.023	0.239	56.4976	0.002	− 0.088	1.652
283.156	2.0125	43.3093	43.2955	− 0.032	0.046	0.232	43.3100	0.002	− 0.050	1.465

The density *ρ*_EOS_ was calculated with the reference equation of state (Span and Wagner 1996), *ρ*_exp_ is the experimental density, and Δ*ρ*/*ρ* = (*ρ*_exp_ − *ρ*_EOS_)/*ρ*_EOS_ is the relative deviation of the experimental density from the calculated density

The expanded uncertainties (*k* = 2) of the measurements are 16 mK for temperature *T* and between (0.1 and 0.7) kPa for pressure *p*

a The dew-point pressure of carbon dioxide at *T* = 283.183 K is *p*_s_ ≈ 4.5059 MPa

**Table 6:** Uncertainty budget for the adsorption capacity *q*_NP_ for carbon dioxide on surface of the sorption sinker and density sinker (see Eq. (16))

	Uncertainty *U* (*k* = 2)	Contribution to *U*_C_(*q*_NP_)/(mmol ·m^−2^) (*k* = 2)	
Source^a^		Sorption sinker	Density sinker
*Our improved gravimetric sorption analyzer*			
Temperature *T*	16 mK	(0.070)	(1.28)
Pressure *p*	0.2 kPa	(0.060)	(1.09)
Density calculated with equation of Span and Wagner (1996)	0.03%	(0.107)	(1.95)
Combined uncertainty in density *U*_C_(*ρ*)^b^	0.043 kg·m^−3^	0.141	2.57
Weighing value m*_CP,vac_ (9.3386 g), m*_S,vac_ (19.6571 g)	60 μg	0.168	0.83
Weighing value m*_CP,fluid_ (9.2117 g), m*_S,fluid_ (19.1868 g)	60 μg	0.168	0.83
Volume of the sorption sinker and the density sinker *V*_CP_ || *V*_S_	0.001% || 0.02%	0.071	0.07
Combined expanded uncertainty (*k* = 2) in adsorption capacity^c^ *U*_C_(*q*_NP_)	0.286		2.83
*A typical commercial gravimetric sorption analyzer*			
Temperature *T*	300 mK	(1.319)	(24.03)
Pressure *p*	3.5 kPa	(0.522)	(9.51)
Density calculated with equation of Span and Wagner (1996)	0.03%	(0.107)	(1.95)
Combined uncertainty in density *U*_C_(*ρ*)^b^	0.055 kg·m^−3^	1.423	25.92
Weighing value m*_CP,vac_ (9.3386 g), m*_S,vac_ (19.6571 g)	80 μg	0.224	1.11
Weighing value m*_CP,fluid_ (9.2117 g), m*_S,fluid_ (19.1868 g)	80 μg	0.224	1.11
Volume of the sorption sinker and the density sinker *V*_CP_ || *V*_S_	0.05% || 0.05%	0.178	3.24
Combined expanded uncertainty (*k* = 2) in adsorption capacity^c^ *U*_C_(*q*_NP_)	1.468		26.17

As an example, the measurement at (*T* = 283.208 K, *p* = 3.9857 MPa, *ρ* = 107.685 kg·m^−3^) of carbon dioxide was taken (see Table 5)

The measured adsorption capacity was significantly smaller than the uncertainty at almost all pressures (see Table 5)

a The influence of the uncertainties of the FTE correction factors *ε*_vac_ and *ε*_fluid_ for the two sinkers, the density of the condensed fluid *ρ*_sorb_, and the geometrical surface area *A* to the uncertainty of *q*_NP_ is relatively small and can be neglected; see Sect. 3.4

b Combined uncertainty according to Eq. (6), which includes the uncertianty in temperature and pressure measurement, and the uncertainty of the density calcuated with the reference equation of Span and Wagner (1996) for carbon dioxide

c Calcuated with Eq. (16)

## Footnotes

1 Certain trade names and products are given to adequately document the experimental equipment and procedures. This does not constitute a recommendation or endorsement of these products by the National Institute of Standards and Technology, nor does it imply that the products are necessarily the best available for the purpose.
